# Prediction of the Fundus Tessellation Severity With Machine Learning Methods

**DOI:** 10.3389/fmed.2022.817114

**Published:** 2022-03-10

**Authors:** Lei Shao, Xiaomei Zhang, Teng Hu, Yang Chen, Chuan Zhang, Li Dong, Saiguang Ling, Zhou Dong, Wen Da Zhou, Rui Heng Zhang, Lei Qin, Wen Bin Wei

**Affiliations:** ^1^Beijing Key Laboratory of Intraocular Tumor Diagnosis and Treatment, Beijing Ophthalmology & Visual Sciences Key Lab, Medical Artificial Intelligence Research and Verification Key Laboratory of the Ministry of Industry and Information Technology, Beijing Tongren Eye Center, Beijing Tongren Hospital, Capital Medical University, Beijing, China; ^2^School of Statistics, University of International Business and Economics, Beijing, China; ^3^School of Banking and Finance, University of International Business and Economics, Beijing, China; ^4^EVision Technology (Beijing) Co. LTD., Beijing, China

**Keywords:** fundus tessellation, fundus tessellated density, fundus tessellation severity, machine learning, the Beijing eye study

## Abstract

**Purpose:**

To predict the fundus tessellation (FT) severity with machine learning methods.

**Methods:**

A population-based cross-sectional study with 3,468 individuals (mean age of 64.6 ± 9.8 years) based on Beijing Eye Study 2011. Participants underwent detailed ophthalmic examinations including fundus images. Five machine learning methods including ordinal logistic regression, ordinal probit regression, ordinal log-gamma regression, ordinal forest and neural network were used.

**Main Outcome Measure:**

FT precision, recall, F1-score, weighted-average F1-score and AUC value.

**Results:**

Observed from the in-sample fitting performance, the optimal model was ordinal forest, which had correct classification rate (precision) of 81.28%, while 34.75, 93.73, 70.03, and 24.82% in each classified group by FT severity. The AUC value was 0.7249. And the F1-score was 65.05%, weighted-average F1-score was 79.64% on the whole dataset. For out-of-sample prediction performance, the optimal model was ordinal logistic regression, which had precision of 77.12% on the validation dataset, while 19.57, 92.68, 64.74, and 6.76% in each classified group by FT severity. The AUC value was 0.7187. The classification accuracy of light FT group was the highest, while that of severe FT group was the lowest. And the F1-score was 54.46%, weighted-average F1-score was 74.19% on the whole dataset.

**Conclusions:**

The ordinal forest and ordinal logistic regression model had the strong prediction in-sample and out-sample performance, respectively. The threshold ranges of the ordinal forest model for no FT and light, moderate, severe FT were [0, 0.3078], [0.3078, 0.3347], [0.3347, 0.4048], [0.4048, 1], respectively. Likewise, the threshold ranges of ordinal logistic regression model were ≤ 3.7389, [3.7389, 10.5053], [10.5053, 13.9323], > 13.9323. These results can be applied to guide clinical fundus disease screening and FT severity assessment.

## Introduction

Although artificial intelligence has been widely applied to image identification (1), speech recognition (2), and natural language processing (3), its impact on medical care is only beginning. Machine learning has been applied to fundus images, optical coherence tomography, and visual field analysis in ophthalmology. It demonstrated excellent classification performance for diabetic retinopathy (4), macular edema (5), glaucoma (6), AMD (7), and retinopathy of prematurity (8). Artificial intelligence mixed with telemedicine has the potential to provide a long-term solution for screening and monitoring patients in primary eye care settings.

The sole straightforward approach to view the choroidal vascular structure under direct vision is fundus tessellation (FT), which is described as the sight of massive choroidal arteries at the posterior fundus pole outside of the peripapillary area (9, 10). Previous research has linked fundus tessellated density (FTD) to age and myopic refractive error, and it has been regarded as one of the most critical early signs of pathological myopia (11, 12). FT has also been linked to several ocular illnesses, including age-related macular degeneration (AMD) (13), choroidal neovascularization (11), central serous chorioretinopathy (14), etc.

Recently, with the development of artificial intelligence image processing technology, computer vision and region of interest (ROI) extraction can effectively identify subtle texture differences that cannot be recognized by human eyes (15). In the past, it has been studied to quantify FT using artificial intelligence methods (16). The purpose of this study is to obtain the threshold range of each grade of FTD by predicting the manual score of FT through machine learning. Fundus photos are commonly used to diagnose disease, but this study is unique in that it uses quantitative data FTD to predict descriptive analysis FT grades, resulting in a reference value range for each grade of FTD. The results can be used to better understand demographic characteristics and long-term follow-up.

## Materials and Methods

### Data Sources

The Beijing Eye Study is a long-term population-based study that began in 2011 (17). It was carried out in five communities in the Haidian district (a northern central Beijing metropolitan area) and three communities in the Daxing district (the village area in the south of Beijing). The study population was examined for the first time in 2011 and for the second time 5 years later in 2016. In the later examination, subjects having choroidal information underwent improved depth imaging spectral-domain optical coherence tomography. The database generated from this study was investigated in detail in the past several years and attracted much attention from the field of ophthalmology. According to the Declaration of Helsinki, the Medical Ethics Committee of the Beijing Tongren Hospital approved the study and all participants gave informed written consent. The ethics committee confirmed that all methods were performed following the relevant guidelines and regulations.

### Participant Selection and Variables for Modeling

The criteria defined for inclusion in this study were aged more than 50 years. All the participants were interview edon standardized questions such as socio-economic characteristics, quality of life, depression, physical activity, known major systemic diseases and quality of vision. They also received a medical and detailed ophthalmological examination. Among a total population of 4,403 participants, 3,468 participants received the examinations, including 1,633 subjects in the rural area and 1,835 subjects in the urban area.

Generally, the same patient will be photographed at the same time with the optic disc and the macula as the center of the fundus, and the fundus map will be divided and graded. For a fundus image with a 45° field of view centered on the optic nerve head, the peripapillary area was divided into 4 quadrants (superior, nasal, inferior, and temporal). The sum of each quadrant's grade is the manual grading of FT, the ratio of the area of the leopard spots to the area of the fundus taken). According to the manual grading results, grading equals to 0 corresponds to no fundus tessellation (FT), grading between 1 and 4 corresponds to light FT, grading between 5 and 8 corresponds to moderate FT, and grading between 9 and 12 corresponds to severe FT. Gender, age, and FTD are employed as major factors in this work to predict FT classification using machine learning methods. Through an artificial intelligence image processing technology, we extracted the exposed choroid from the fundus, and then calculated the average exposed choroidal area per unit fundus area, which is called FTD (16).

Before statistical analysis, we removed the missing data, outliers of the selected variables, and duplicate data based on the ID and Eye indicators. Finally, 3,419 samples were included in our dataset.

### Statistical Analysis

In this study, all statistical analyses were performed on R software version 4.1.2. Descriptive statistics and machine learning methods were employed for data analysis. The *P* < 0.05 was considered statistically significant.

Descriptive statistical analyses were conducted separately for a classified group by manual grading of FT (0, 1 ~ 4, 5 ~ 8, 9 ~ 12), means and standard deviation (SD) were reported for FTD and age, sample size was reported for gender. We used five machine learning methods including ordinal logistic regression, ordinal probit regression, ordinal log-gamma regression, ordinal forest and neural network to fit our dataset, and calculated the in-sample correct precision, recall, F1-score, and weighted-average F1-score on the whole dataset to assess the goodness of fit. In the process of model training, we introduced the square term of FTD to add the non-linear effect. To perform the out-of-sample validation, we randomly assigned 50% of the dataset as a training set and the remaining 50% of the dataset as the validation set, and reported the precision, recall, F1-score and weighted-average F1-score on the validation set. Then, the receiver operating characteristic (ROC) curve and the area under the curve (AUC) were employed to identify the accuracy of the five machine learning methods (18).

## Machine Learning Methods

### Ordinal Regression

The ordinal regression models are the extensions of binomial regression, and are also called ranking learning in machine learning. These models are used to predict the dependent variable with ordered multiple categories (19). Suppose the underlying process to be characterized is


$$\begin{array}{l} Y^{*}=X^{T}\beta +\varepsilon , \end{array}$$

where Y^*^ is the exact but unobserved dependent variable; *X* is the vector of independent variables, ε is the error term, and β is the vector of regression coefficients that we wish to estimate. Further suppose that we cannot observe Y^*^, we instead can only observe the categories of response


$$Y=\{\begin{array}{ccc} 0 & if & Y^{*}\leq \mu_{1}\\ 1 & if & \mu_{1}<Y^{*}\leq \mu_{2}\\ 2 & if & \mu_{2}<Y^{*}\leq \mu_{3}\\ \vdots & \vdots & \vdots \\ K & if & \mu_{K}<Y^{*} \end{array}$$

where the parameters μ_*i*_(*i* = 1, ⋯, *K*) are the externally imposed endpoints of the observable categories. Then the ordered regression techniques will use the observations *Y*, which are a form of censored data on Y^*^ to fit the parameter vector β.

To estimate the coefficients in models, we need to choose a link function for defining cumulative probabilities, such as logit, probit, and log-gamma link function, and the corresponding models are ordinal logistic regression, ordinal probit regression, and ordinal log-gamma regression.

### Ordinal Forest

The ordinal forest (OF) method allows ordinal regression with high-dimensional and low-dimensional data. After having constructed an OF prediction rule using a training dataset, it can be used to predict the values of the ordinal target variable for new observations (20). Moreover, using the (permutation-based) variable importance measure of OF, it is also possible to rank the covariates concerning their importance in the prediction of the values of the ordinal target variable. The concept and assumption of OF are similar to ordinal regression. The main idea of OF is to optimize score values μ_*i*_(*i* = 1, ⋯, *K*) to be used in place of the class values 1, ⋯, *K* of the ordinal target variable in standard regression forests by maximizing the out-of-bag (OOB) prediction performance measured by a performance function.

### Neural Networks

In this study, we used the neural network algorithm provided by the monmlp package. The monmlp package uses neural networks to predict ordinal response variables, it implements one and two hidden-layer multi-layer perceptron neural network (MLP) models. An optional monotone constraint, which guarantees monotonically increasing behavior of model outputs concerning specified covariates, can be added to the MLP. The resulting monotone MLP (MONMLP) regression model is based on Zhang and Zhang (21). Early stopping can be combined with bootstrap aggregation to control over-fitting. The model reduces to a standard MLP neural network if the monotone constraint is not invoked. In this paper, we have constructed a two-layer neural network, each layer contains eight neurons, the hidden layer transfer function is “tansig,” output layer transfer function is “linear,” and the optimx optimization method is “BFGS.”

## Results

Table 1 showed that the study population consisted of 3,419 participants [1,445 male (42.26%) and 1,974 females (57.74%)], the mean age was 53.55 (SD = 9.51); the mean of FTD was 0.18 (SD = 0.09). The light FT group had the largest sample size [2,312 participants (67.62%)], the remaining three groups no FT, Moderate FT, and Severe FT containing 282 [8.25%], 684 [20.01%], and 141 [4.12%] participants, respectively. With the increase of FT severity, the FTD shows an upward trend, the mean FTD of no FT group was 0.07 (SD = 0.04), while the mean FTD of severe FT group reached 0.33 (SD = 0.06). The upward trend was also observed for age, the mean age of no FT group was 50.45 (SD = 8.30), while the mean age of severe FTgroup reached 65.04 (SD = 7.21). In the no FT group and light FT group, there were more females than males, but in the moderate FT group and severe FT group, there were more males than females.

**Table 1:** Descriptive statistics of the original sample.

**Manual grading of FT**		**FTD**	**Age**	**Gender**	
**Attribute**	**Size *N* (%)**	**Mean (SD)**	**Mean (SD)**	**Male *N* (%)**	**Female *N* (%)**
No FT	282 (8.25)	0.07 (0.04)	50.45 (8.30)	85 (5.88)	197 (9.98)
Light FT	2,312 (67.62)	0.15 (0.07)	51.43 (8.70)	919 (63.60)	1,393 (70.57)
Moderate FT	684 (20.01)	0.28 (0.07)	59.66 (8.55)	366 (25.33)	318 (16.11)
Severe FT	141 (4.12)	0.33 (0.06)	65.04 (7.21)	75 (5.19)	66 (3.34)
Total	3,419	0.18 (0.09)	53.55 (9.51)	1,445	1,974

Table 2 summarized the model fitting results for each method. For Ordinal logistic regression, Ordinal probit regression and Ordinal log-gamma regression, the coefficients of age, gender, FTD, and FTD squared are all statistically significant. The threshold 1 ~ 4 in Table 2 represented the threshold of $Y^{*}=X^{T}\hat{\beta }$, which corresponds to the four types of FT. For example, in the ordinal logistic regression, if Y^*^ <3.7389, the corresponding result of fundus tessellation grading should be no FT group. The importance measures of each variable were reported for the ordinal forest algorithm, among which FTD was the most important variable.

**Table 2:** The results of different algorithms.

	**Ordinal logistic regression**	**Ordinal probit regression**	**Ordinal log-gamma regression**	**Ordinal forest**	**Neural network**
	**Coefficient (SE)**	**Coefficient (SE)**	**Coefficient (SE)**	**Importance**	
Age	0.0575*** (0.0051)	0.0330*** (0.0027)	0.0317*** (0.0028)	0.0216	–
Gender	−0.2026* (0.0889)	−0.0964* (0.0481)	−0.0835* (0.0488)	0.0001	–
FTD	35.6489*** (2.4007)	17.2249*** (1.1994)	17.1730*** (1.1980)	0.2327	–
FTD^2^	−24.4600*** (5.0442)	−9.5187*** (2.5911)	−8.0721** (2.6208)	0.2090	–
Threshold 1 (no FT)	≤ 3.7389	≤ 1.9527	≤ 2.0456	[0, 0.3078]	–
Threshold 2 (light FT)	[3.7389, 10.5053]	[1.9527, 5.5435]	[2.0456, 5.6847]	[0.3078, 0.3347]	–
Threshold 3 (moderate FT)	[10.5053, 13.9323]	[5.5435, 7.4299]	[5.6847, 7.6598]	[0.3347, 0.4048]	–
Threshold 4 (severe FT)	>13.9323	>7.4299	>7.6598	(0.4048, 1)	–

***P ≤ 0.001; **P ≤ 0.01; *P ≤ 0.05.

Tables 3, 4 presented the in-sample and out-of-sample precision, recall, F1-score and weighted-average F1-score for each machine learning method. Accuracy was a metric for classification models that measures the number of predictions that are correct as a percentage of the total number of predictions that are made. It was a useful metric only when you have an equal distribution of classes on your classification. As can be seen from Table 1, the sample in this paper was unbalanced. Precision and Recall were two most common metrics that take into account class imbalance. They were also the foundation of the F1 score. The goal of the F1 score was to combine the precision and recall metrics into a single metric (7, 22). At the same time, the F1 score has been designed to work well on imbalanced data, which taken into account not only the number of prediction errors that your model makes, but that also look at the type of errors that are made. Then we weighted the F1-score of each class by the number of samples from that class to get the weighted-average F1-score.

**Table 3:** In-sample correct classification rate for each machine learning method.

	**Attribute**	**Ordinal logistic regression**	**Ordinal probit regression**	**Ordinal log-gamma regression**	**Ordinal forest**	**Neural networks**
Precision	No FT	0.1950	0.1312	0.1596	0.3475	0.1986
	Light FT	0.9208	0.9299	0.9330	0.9373	0.9252
	Moderate FT	0.6535	0.6477	0.6360	0.7003	0.6974
	Severe FT	0.0851	0.0922	0.0709	0.2482	0.3262
	Total	0.7730	0.7730	0.7742	0.8128	0.7950
Recall	No FT	0.5556	0.5692	0.5921	0.7101	0.5657
	Light FT	0.8217	0.8162	0.8158	0.8458	0.8281
	Moderate FT	0.6340	0.6392	0.6416	0.7065	0.6964
	Severe FT	0.5000	0.4815	0.4762	0.8537	0.8846
F1-score		0.5334	0.5240	0.5254	0.6505	0.6236
Weighted-average F1-score		0.7458	0.7406	0.7422	0.7964	0.7743

**Table 4:** Out-of-sample correct classification rate for each machine learning method.

	**Attribute**	**Ordinal logistic regression**	**Ordinal probit regression**	**Ordinal log-gamma regression**	**Ordinal forest**	**Neural networks**
Precision	No FT	0.1957	0.1667	0.1739	0.2174	0.2681
	Light FT	0.9268	0.9312	0.9312	0.9083	0.9065
	Moderate FT	0.6474	0.6419	0.6281	0.6364	0.5604
	Severe FT	0.0676	0.0676	0.0676	0.1081	0.1892
	Total	0.7712	0.7706	0.7683	0.7601	0.7503
Recall	No FT	0.4821	0.4694	0.4706	0.3846	0.4111
	Light FT	0.8128	0.8098	0.8080	0.8085	0.8114
	Moderate FT	0.6657	0.6676	0.6647	0.6696	0.6559
	Severe FT	0.7143	0.7143	0.6250	0.6667	0.3333
F1-score		0.5446	0.5382	0.5293	0.5376	0.5145
Weighted-average F1-score		0.7419	0.7390	0.7371	0.7367	0.7332

Observed from the in-sample fitting performance, the optimal model was Ordinal forest, which had a weight-F1-score of 79.64%, F1-score of 65.05%, and precision of 81.28% on the whole dataset. The precision in each classified group by FT severity were 34.75, 93.73, 70.03, and 24.82%, while the recall were 71.01, 84.58, 70.65, and 85.37%. For out-of-sample prediction performance, the optimal model was Ordinal logistic regression, which had a weighted-average F1-scoreof 74.19%, a F1-score of 54.46% and a correct classification rate of 77.12% on the validation dataset. The precision in each classified group by FT severity were 19.57, 92.68, 64.74, and 6.76%, while the recall were 48.21, 81.28, 66.57, and 71.43%. It can also be seen that obvious differences existed in the correct classification rate of different FT groups, the light FT group had the highest correct classification rate, while the severe FT group was the lowest.

**Figure 1:**
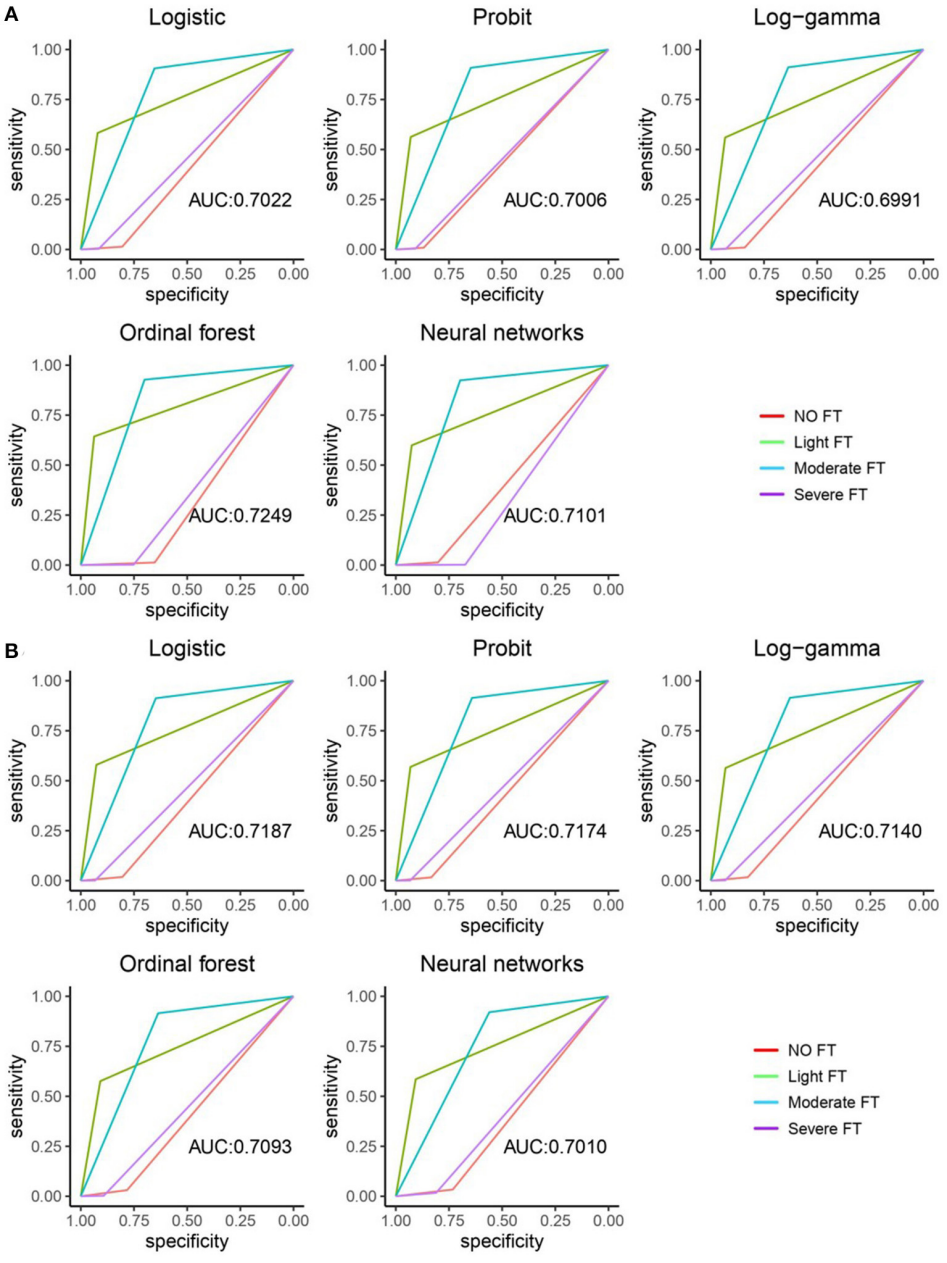
ROC curve and AUC value of five machine learning methods. **(A)** The in-sample ROC curves and AUC values of the five machine learning methods. **(B)** The out-sample ROC curves and AUC values of five machine learning methods.

The ROC curve and AUC value of five machine learning methods were shown in Figure 1. Figure 1A showed in-sample performance and Figure 1B showed out-of-sample performance. From a single picture front, the light FTgroup and moderated FT group had better classification performance, in comparison, the classification performance of no FT group and severe FT group is poor. The values in the figure represented the weighted average area under the four curves (AUC), which was a generalized measure of multi-classification performance, weight was the reciprocal of the sample size of each class. The AUC value did not change drastically in the out-of-sample, which indicated that the performance of the five machine learning methods was relatively stable. Comparatively, observed from the in-sample fitting performance, the optimal model was Ordinal forest, the AUC value was 0.7249. For out-of-sample prediction performance, the optimal model was Ordinal logistic regression, which AUC value was 0.7187.

Although the five methods showed relatively similar out-of-sample model performance metrics, we preferred the result of Ordinal logistic regression to give the following risk scheme to predict the severity of FT by age, gender and FTD from patients.


$$\begin{array}{l} Y^{*}=0.0\text{575}Age-0.\text{2026 }Gender+\text{35.6489 }FTD\\ \;-\text{24.4600 }FTD^{2}\\ Y=\{\begin{array}{ccc} NoFT & if & Y^{*}\leq \text{3.7389}\\ LightFT & if & \text{3.7389}<Y^{*}\leq \text{10.5053}\\ ModerateFT & if & \text{10.5053}<Y^{*}\leq 1\text{3.9323}\\ SevereFT & if & Y^{*}>\text{13.9323} \end{array} \end{array}$$

## Discussion

As far as we are aware, this work is the first to forecast the FT classification using machine learning technology, and is also the first to give the FTD reference value range of FT classification. This study obtained the value of FTD through an artificial intelligence image processing technology, verified the accuracy of machine learning in predicting FT classification, provides 5 machine learning methods with high accuracy, and obtained the thresholds of 4 FT classification in each machine learning classification methods except the neural network method. Our findings suggest that observed from the in-sample fitting performance, the optimal model was ordinal forest. For out-of-sample prediction performance, the optimal model was ordinal logistic regression. The FTD reference value range generated in this study can be used for guiding clinical fundus disease screening and FT severity assessment.

Estimates of the severity of FT are crucial, but they can also be perplexing in the absence of a recognized objective marker of severity. We used five machine learning methods to fit our data set in this cohort study, including ordinal logistic regression, the ordinal probit regression, the ordinal log-gamma regression, ordinal forest and neural network, calculated the in-sample precision, recall, F1-score, weighted-average F1-score and AUC value to evaluate the goodness of fit, and reported the same model performance metrics of out-of-sample validation. Previous studies have reported that FT is related to age and gender (9). In our model, age, gender, and FTD were introduced as variables. At the same time, to improve the non-linear effect, we also introduced the square term of FTD. The coefficients of age, gender, FTD, and FTD squared were all statistically significant in Ordinal logistic regression, Ordinal probit regression, and Ordinal log-gamma regression. For the ordinal forest algorithm, the important measures of each variable were presented, with FTD being the most important variable and gender being the least important. From the observation of fitting performance within the sample, the optimal model was ordinal forest, and the correct classification rate on the whole data set was 81.28%, the F1 score was 65.05%, the weighted-average F1-score was 79.64%. For out-of-sample prediction performance, the best model was Ordinal logistic regression, and its correct classification rate on the validation dataset was 77.12%, the F1 score was 54.46%, the weighted-average F1-score was 74.19%. Overall, the classification accuracy of different FT groups differed significantly (Tables 3, 4). The light FT group had the best categorization precision, while the severe FT group has the lowest.

The low classification precision of severe FT could be a result of extensive or patchy chorioretinal atrophy (11, 23) in fundus pictures, which impairs FTD accuracy. Additionally, because this study is directed at those over the age of 50 when the degree of FT is severe, patients are more likely to be complicated with other eye diseases (24), which may introduce additional bias. Increasing the number of independent samples, repeating the model's training process and verifying the results may increase the model's accuracy. Furthermore, this large-scale cohort study based on the elderly population gathered detailed eye and whole-body data, including axis length, diopter, weight, and other characteristics that may be linked to FTD (16). Various research on FT risk variables might be added to this foundation. This cohort, for example, might aid in the development of a more accurate pathological myopia grading model.

In conclusion, we developed a valid approach to predict the severity classification of FT by using consistent clinician evaluation and machine learning, so as to obtain the threshold of each classification of FTD, which is expected to become the reference value of clinical FTD. FTD can be used to evaluate the severity of fundus lesions directly, and improve the treatment of chronic fundus diseases. We know that deep learning, such as convolutional neural network, can also predict FT classification through images, but so far, there is no research to compare its accuracy with machine learning methods. We think this may be the future research direction. Further, we should try modifying the data and repeating the training procedure with more samples to improve these outcomes. Additionally, while ordinal logistic regression is the most advanced technique commonly employed in biomedical data research, other technologies, such as deep learning, can be applied to enhance the quality of the produced results.

Potential limitations should be mentioned. First, in the Beijing Eye Study 2011, disparities between participants and non-participants may have resulted in a selection artifact with a reasonable response rate of 78.8%. Second, for the population-based study, we enrolled all eligible subjects from the study region. As a result, illnesses may have influenced FTD, particularly about choroidal thickening or thinning. Third, this study is not a random sample from the Chinese population. This may affect the applicability of the model trained on our data to other medical institutions.

## Conclusions

We applied machine learning to collect FTD data from the Beijing Eye Study, evaluated the accuracy of FT classification, and obtained the threshold range of each grade of FTD. Our findings suggest that observed from the in-sample fitting performance, the optimal model was ordinal forest, which the threshold ranges of no FT, light, moderate, and severe FT were [0, 0.3078], [0.3078, 0.3347], [0.3347, 0.4048], [0.4048, 1]. For out-of-sample prediction performance, the optimal model was ordinal logistic regression, which had the threshold ranges of no FT, light, moderate, and severe FT were ≤ 3.7389, [3.7389, 10.5053], [10.5053, 13.9323], >13.9323, respectively. These results can be applied to guide clinical fundus disease screening and FT severity assessment.

## Data Availability Statement

The original contributions presented in the study are included in the article/supplementary material, further inquiries can be directed to the corresponding author/s.

## Author Contributions

LS, WW, and LQ: design of the study. XZ, TH, and YC: development of the algorithm. LS, CZ, LD, WZ, and RZ: gathering the data. LS, XZ, TH, YC, SL, and ZD: performing the data analysis. LS and CZ: drafting the first version of the manuscript. All authors: revision and approval of the manuscript. All authors contributed to the article and approved the submitted version.

## Funding

This study was supported by National Natural Science Foundation of China (No. 82000916), the Priming Scientific Research Foundation for the junior researcher in Beijing Tongren Hospital, Capital Medical University (2016-YJJ-ZLL-009), Beijing Hospitals Authority Youth Programme, code: QML20180204, the Priming Scientific Research Foundation for the junior researcher in Beijing Tongren Hospital, Capital Medical University (No. 2018-YJJ-ZZL-045). Dongcheng District Outstanding Talent Nurturing Program (2020-dchrcpyzz-42), the Fundamental Research Funds for the Central Universities in UIBE (CXTD10-10), and University of International Business and Economics (UIBE) Huiyuan distinguished young scholars research fund (Grant 20JQ07).

## Conflict of Interest

SL and ZD are employed by EVision Technology (Beijing) co. LTD., Beijing, China. The remaining authors declare that the research was conducted in the absence of any commercial or financial relationships that could be construed as a potential conflict of interest.

## Publisher's Note

All claims expressed in this article are solely those of the authors and do not necessarily represent those of their affiliated organizations, or those of the publisher, the editors and the reviewers. Any product that may be evaluated in this article, or claim that may be made by its manufacturer, is not guaranteed or endorsed by the publisher.
